# Predictors of Functional and Quality of Life Outcomes following Deep Brain Stimulation Surgery in Parkinson's Disease Patients: Disease, Patient, and Surgical Factors

**DOI:** 10.1155/2017/5609163

**Published:** 2017-08-09

**Authors:** Hesham Abboud, Gencer Genc, Nicolas R. Thompson, Srivadee Oravivattanakul, Faisal Alsallom, Dennys Reyes, Kathy Wilson, Russell Cerejo, Xin Xin Yu, Darlene Floden, Anwar Ahmed, Michal Gostkowski, Ayman Ezzeldin, Hazem Marouf, Ossama Y. Mansour, Andre Machado, Hubert H. Fernandez

**Affiliations:** ^1^Center for Neurological Restoration, Cleveland Clinic, 9500 Euclid Avenue, Mail Code U2, Cleveland, OH 44195, USA; ^2^Case Western Reserve University, University Hospitals of Cleveland, 11100 Euclid Avenue, Cleveland, OH 44106, USA; ^3^Department of Neurology, Alexandria University, El Hadara University Hospital, El Hadara Kebly, Alexandria, Egypt; ^4^Department of Quantitative Health Sciences, Cleveland Clinic, 9500 Euclid Avenue, Mail Code JJN3-01, Cleveland, OH 44195, USA; ^5^Neurological Institute, Center for Outcomes Research and Evaluation, Cleveland Clinic, 9500 Euclid Avenue, Cleveland, OH 44195, USA; ^6^Department of Neurology, Cleveland Clinic Florida, 2950 Cleveland Clinic Blvd, Fl 3, Weston, FL 33331, USA

## Abstract

**Objective:**

The primary objective was to evaluate predictors of quality of life (QOL) and functional outcomes following deep brain stimulation (DBS) in Parkinson's disease (PD) patients. The secondary objective was to identify predictors of global improvement.

**Methods:**

PD patients who underwent DBS at our Center from 2006 to 2011 were evaluated by chart review and email/phone survey. Postoperative UPDRS II and EQ-5D were analyzed using simple linear regression adjusting for preoperative score. For global outcomes, we utilized the Patient Global Impression of Change Scale (PGIS) and the Clinician Global Impression of Change Scale (CGIS).

**Results:**

There were 130 patients in the dataset. Preoperative and postoperative UPDRS II and EQ-5D were available for 45 patients, PGIS for 67 patients, and CGIS for 116 patients. Patients with falls/postural instability had 6-month functional scores and 1-year QOL scores that were significantly worse than patients without falls/postural instability. For every 1-point increase in preoperative UPDRS III and for every 1-unit increase in body mass index (BMI), the 6-month functional scores significantly worsened. Patients with tremors, without dyskinesia, and without gait-freezing were more likely to have “much” or “very much” improved CGIS.

**Conclusions:**

Presence of postural instability, high BMI, and worse baseline motor scores were the greatest predictors of poorer functional and QOL outcomes after DBS.

## 1. Introduction

 Although deep brain stimulation surgery (DBS) has been established as a superior treatment option for advanced Parkinson's disease (PD) [1], there has been a discrepancy between motor and functional/quality of life (QOL) outcomes after surgery [2, 3]. While motor outcomes are believed to improve significantly in the majority of patients following DBS compared to medical therapy alone [2], QOL outcomes are not as consistent with only about 50% of patients showing some improvement in QOL after surgery [3]. This has led to a recent shift of focus in DBS research from motor outcomes to functional and QOL outcomes. In recent years, an increasing number of studies attempted to find new clinical predictors of these outcomes to complement or replace traditional motor predictors with the goal of ultimately translating into better selection of surgical candidates [4–7]. In addition to commonly studied factors such as age and disease duration, our group and others explored less conventional outcome predictors like socioeconomic status [8], mood and psychosocial factors [3, 9], and preoperative cognitive patterns [10]. In this study, we look at the effect of several disease, patient, and surgical factors on QOL, functional, and global measures.

## 2. Methods

We performed a retrospective review of consecutive PD patients who underwent DBS implantation (subthalamic or internal globus pallidus) at our center from 2006 to 2011 and had near-complete charting. We collected two health status measures (HSM), the European Quality of Life 5-Dimension Questionnaire (EQ-5D) and the Unified Parkinson's Disease Rating Scale, part 2: activities of daily living (UPDRS II) [the Movement Disorders Society-Unified Parkinson's Disease Rating Scale or MDS-UPDRS II was used for visits after 2008], for the following time points when available: latest preoperatively (within one month prior to surgery), 6 months postoperatively (range: 3–9 months), and 12 months postoperatively (range: 9–15 months). The EQ-5D is a standardized instrument for measuring health-related QOL in terms of five dimensions (5D), mobility, self-care, usual activities, pain/discomfort, and anxiety/depression, producing a single index value for overall health status. In addition, we also conducted a one-item Patient Global Impression of Change Scale (PGIS) via phone/email survey using an IRB-approved phone/email script for all study subjects to provide additional long-term global outcome specific for this study. The PGIS aims at determining the patient's global impression of his/her current state compared to the state prior to DBS surgery with the following possible answers: very much improved, much improved, minimally improved, no change, minimally worse, much worse, or very much worse. The PGIS survey was distributed in mid-2011 (1 to 5 years from date of first surgery). To match our patient-perceived outcomes to clinicians' perception of overall outcome after surgery, we also conducted a one-item Clinician's Global Impression of Change Scale (CGIS) survey for study subjects. The CGIS aims at determining the clinician's impression of the overall clinical change in each patient after surgery using the same 7-point anchor as the PGIS. The CGIS scores were retrospectively determined based on the full information derived from patients' medical records and postoperative office visits during the same time periods when the PGIS was obtained.

The following potential clinical predictors were collected for all patients from their preoperative visits and operative reports:

- Disease factors: disease duration, dopaminergic burden (based on levodopa equivalent daily dose [LEDD] conversion), preoperative UPDRS part III motor subscale (MDS-UPDRS part III after 2008) in the ON state, presence of tremors, dyskinesia, freezing of gait (FOG), and falls/balance dysfunction. Clinical symptoms were based on the patients' major complaints when presenting for DBS evaluation. Although these complaints were matched to their UPDRS III/MDS-UPDRS III subscores on exam, no formal score cutoffs were used for quantification. This was based, in part, on the difficulty of developing unified cutoff scores for the two different versions of the motor scale. More importantly, since this study was geared towards patients' experience, we meant to put more emphasis on patient-reported symptoms rather than motor subscores as potential predictors of QOL and functional outcomes
- Patient factors: age, marital status, and body mass index (BMI)
- Surgical factors: surgery type (i.e., unilateral, staged bilateral, or simultaneous bilateral) and number of intraoperative microelectrode passes

### 2.1. Statistical Analysis

To determine short-term and intermediate predictors of improved functional state and QOL, we created simple linear regression models where the 6-month and 12-month postoperative UPDRS II/MDS-UPDRS II score or EQ-5D index was the dependent variable. For each of these models, we adjusted for the preoperative score by including it in the model as a covariate. For each of the clinical predictors listed in Methods, we created a separate model where that predictor was the independent variable. The effect of each predictor on outcome is provided through estimated beta coefficients and associated 95% confidence intervals. Patients with missing data for certain time point were not included in the analysis for that time point.

To determine predictors of global outcomes based on patient's and clinician's perceptions, we dichotomized the responses in the PGIS and CGIS into “much improved” or “very much improved” versus all other responses. For categorical predictors, we computed the proportion and percent of patients with PGIS or CGIS of “much improved” or “very much improved.” Fisher's exact tests were used to determine statistical significance. For continuous predictors, we created logistic regression models. We estimated odds ratios and computed 95% confidence intervals for each. Due to the exploratory nature of this study, we did not correct for multiple comparisons.

All analyses were conducted using R, version 3.0.1, and *P* values less than 0.05 were considered statistically significant. This study was approved by Cleveland Clinic's institutional review board.

## 3. Results

### 3.1. Predictors of Functional and QOL Outcomes

There were 130 patients in the dataset. Overall, patients had an average age at time of surgery of 63.0 (±9.1) years, had PD for 10.7 (±5.1) years, had an average BMI of 27.5 (±5.2) kg/m^2^, and had an average LEDD of 1190 (±666). The cohort was more predominantly male (70.8%), white (86.9%), and married (66.9%). Of the 130 patients, 55 (42.3%) had unilateral surgery, 50 (38.4%) had bilateral staged surgery, and 25 (19.2%) had bilateral unstaged surgery. Most patients were implanted in the STN, 124 (95.3%).

Forty-five patients had both preoperative and postoperative data at 6 months and at 12 months. This group had mostly similar characteristics to the group with incomplete data except for having a younger average age (60.4 years, *P* = 0.019). Of these 45 patients, 29 patients (64.4%) had bilateral surgery. At 6 months, statistically significant improvement was seen for both the mean EQ-5D index (*P* = 0.03) and the average UPDRS II/MDS-UPDRS II score (*P* = 0.002). However, one year after surgery, no significant improvement or worsening was found for either scale.

There were 116 patients for which the CGIS could be completed from the available records. Of these, 19 (16.4%) were rated as “very much improved,” 63 (54.3%) as “much improved,” 23 (19.8%) as “minimally improved,” 6 (5.2%) as “no change,” 3 (2.6%) as “minimally worsened,” and 2 (1.7%) as “much worsened.”

There were 67 patients that completed the PGIS. Of these 67 patients, 29 (43.3%) reported “very much improved,” 25 (37.3%) reported “much improved,” 10 (14.9%) reported “minimally improved,” 2 (3.0%) reported “much worse,” and 1 (1.5%) reported “very much worse.”

Table 1 displays results of the simple linear regression models relating different predictors to approximate 6-month and 1-year HSM. Patients that had falls/balance-dysfunction had 6-month mean UPDRS II/MDS-UPDRS II scores that were 6.48 points worse than patients that did not have falls/balance-dysfunction (*P* = 0.019). A similar estimated effect of falls/balance-dysfunction was found at the 1-year UPDRS II/MDS-UPDRS II scores, but statistical significance was not achieved (Estimated effect = 6.45; *P* = 0.0634). Similarly, patients that had falls/balance dysfunction at baseline had 1-year mean EQ-5D index scores that were 0.12 points lower than patients who did not have falls/balance dysfunction (*P* = 0.019) but this effect was not significant at 6 months (*P* = 0.2690). After adjusting for preoperative EQ-5D index, for every one-point increase in the preoperative UPDRS III/MDS-UPDRS III score in the ON state, the 6-month EQ-5D index worsened by 0.01 units (*P* = 0.005). However, no relationship between UPDRS III/MDS-UPDRS III score in the ON state and the EQ-5D index was seen at 1 year. Moreover, no relationship was found between the UPDRS III/MDS-UPDRS III and the UPDRS II/MDS-UPDRS II scores at either 6 months or 1 year.

The BMI distribution in the patient group was as follows: underweight, 0 patients; normal weight, 12 patients; overweight, 16 patients; and obese, 15 patients. After excluding 2 outliers, the estimated effect of BMI on 6-month UPDRS II/MDS-UPDRS II score was significant (*P* = 0.033). For every one-unit increase in BMI, the UPDRS II/MDS-UPDRS II score at 6 months worsened by 0.49 points on average. There was also evidence of a similar but weaker association at 1 year (*P* = 0.0507). However, at both six months and one year after surgery, no significant associations were found between BMI and EQ-5D index.

### 3.2. Predictors of Global Outcomes

Table 2 displays the results relating categorical predictors to the PGIS and CGIS. While the majority of the patients in our cohort were rated to be “very much” or “much” improved in the PGIS and CGIS, of patients that had tremor, 84.0% showed “much” or “very much” improvement on the CGIS, whereas only 60.0% of patients without tremor showed “much” or “very much” improvement (*P* = 0.0075). Patients without dyskinesia and patients without freezing were more likely to show “much” or “very much” improvement on the CGIS (*P* = 0.038 and *P* = 0.0315, resp.). There were no statistically significant results when correlating continuous predictors to the CGIS and the PGIS. There was also no correlation between global outcomes and the cognitive and mood predictors included in our previously reported cognitive study [10].

### 3.3. Noninfluential Factors

There was no significant association between QOL, functional, or global outcomes and patients' age, disease duration, laterality of surgery (unilateral versus bilateral), number of intraoperative electrode passes, LEDD, or marital status. However, some interesting trends were observed including a trend between shorter disease duration and more improvement in EQ-5D index at 1 year (*P* = 0.0696) and a PGIS of “much” or “very much improvement” (*P* = 0.0683). There was also a trend between higher number of intraoperative microelectrode passes on the left and less improvement of EQ-5D index at 1 year (*P* = 0.0508).

## 4. Discussion

In this study, we looked at predictors of functional and QOL outcomes of DBS in a cohort of PD patients who underwent DBS under a standardized protocol. We explored a large number of potential predictors including several disease, patient, and surgical factors. We have previously reported the socioeconomic and cognitive data of the same cohort [8, 10]. In the current study, we found that the baseline presence of falls/balance dysfunction was associated with worse 6-month functional outcome after DBS with a trend towards a similar poor outcome at 1 year after surgery. Falls/balance dysfunction were also predictive of poor QOL outcome at 1 year. In addition, the presence of FOG and absence of tremors, other indicators of predominantly axial disease, predicted poorer CGIS. These relationships are in agreement with findings by Welter and colleagues who reported poor functional outcomes 6 months after surgery in patients with axial motor symptoms preoperatively [4]. On the same note, Maier and colleagues reported an association between higher axial motor score and worse subjective perceived outcome after DBS [11]. Patients with predominantly axial disease are known to attain less motoric benefit from DBS [12] and our results suggest that this might extend into functional, QOL, and global outcomes after surgery.

The presence of dyskinesia preoperatively was associated with somewhat poorer long-term global outcome in our study as represented by the CGIS. In 2011, Daniels and colleagues reported similar findings showing that patients with lower preoperative dyskinesia scores did better on QOL measures after surgery as represented by the Parkinson's Disease Questionnaire-39 (PD-Q39) and the 36-Item Short Form Health Survey (SF-36) [5]. Although the presence of dyskinesia is considered a classical indication for DBS and patients often experience reduction of dyskinesia after surgery especially when the dose of levodopa is successfully reduced [13], this does not necessarily translate into improvement in QOL or global perceivable outcome [5]. It is well known that, in many occasions, dyskinesia is more bothersome to patients' families than the patients themselves and is not detrimental to the QOL of PD patients [14]; in addition, the loss of levodopa peak-dose euphoria after dose reduction postoperatively may explain why patients with preoperative dyskinesia report less improvement in QOL after surgery when their dyskinesia improves as suggested by Daniels and colleagues [5]. Other possible explanations include the fact that the presence of dyskinesia, in general, indicates more advanced disease and that some patients may rarely experience worsening dyskinesia with stimulation [15].

Our results agreed with both Welter's and Daniels' studies in confirming a role for preoperative UPDRS III motor score in predicting functional/QOL outcomes following DBS, with higher scores indicating worse outcomes, perhaps as a general indication of more advanced disease [4, 5]. Soulas and colleagues confirmed the finding by Welter which demonstrates that age and disease duration are predictors of poorer outcome after surgery [6], but these factors were noninfluential in our study, although longer disease duration showed a weak trend towards poorer EQ-5D and PGIS in our group. In a study by Floden and colleagues from our group utilizing a different QOL scale (PDQ-39), preoperative episodic memory, depression, and bilateral surgery were the most influential predictors [3].  Table 3 displays a summary of the studies that looked at predictors of functional, QOL, and global DBS outcomes since the early 2000s.

In addition to disease characteristics, our study suggests that certain patient characteristics, regardless of disease severity, may also influence functional and QOL outcomes after DBS. In addition to the impact of socioeconomic status, which we previously reported [8], BMI seems to have a similar effect on DBS outcomes. Higher preoperative BMI predicted worse functional outcomes at 6 months and, to a lesser extent, at 1 year after surgery. This finding could be another reflection of poorer socioeconomic status where obesity is more prevalent [16] but it may also be related to further weight gain incurred after surgery. Weight gain after DBS has been frequently reported in literature and is thought to be secondary to reduction in the metabolic rate after resolution of tremor/dyskinesia and/or a direct stimulation effect on appetite centers [17–20]. Adding more weight after DBS in patients who are already overweight or obese can translate into patient perception of a suboptimal functional outcome. A post hoc analysis of our patient group revealed that the BMI increased in 55% of the patients at 1 year after surgery with an increment higher than 1 kg/m^2^ in 35% and higher than 2 kg/m^2^ in 17%. In a recent study, preoperative obesity was associated with poor axial and cognitive outcomes after DBS [21] but our study is the first to test the effect of BMI on functional and QOL outcomes. This is an important area that warrants further study. Exploring the role of dieting and/or exercise prior to DBS on motor and nonmotor outcomes may be of value.

There are several limitations to our study. In addition to the retrospective nature of the study, the sample size was fairly small for the number of comparisons and the study may have been underpowered, especially for the functional and QOL outcomes. Nonetheless, the demographic features of the subset of patients with complete data versus the entire cohort showed largely similar demographics; therefore, we believe that this subset still represented the PD population who underwent DBS surgery. The slight difference in age between the two groups is probably attributed to the fact that younger patients are more familiar with technology and therefore more likely to complete computer-based surveys and assessment scales. Further studies utilizing larger patient cohorts are needed to better study predictors of functional and QOL outcomes following DBS. We did not look into other QOL measures that are more specific for PD such as the PDQ-39 due to limited availability of data in this cohort; however, PDQ-39 data were available in a more recent patient cohort and were recently published by our group in a separate paper as discussed earlier [3]. Also we did not study functional/QOL outcomes beyond 1 year after surgery which, although consistent with other similar studies, does not account for how the benefit from surgery holds up against disease progression over the years. The absence of statistically significant difference in QOL and functional scores one year after surgery compared to preoperative scores was inconsistent with the results of previous DBS randomized trials [22]. However, the majority of our patients rated their overall global outcome as much or very much improved on the PGIS survey that was distributed to the patients 1 to 5 years after the date of surgery. This indicates that DBS still exerted a very positive impact on patients' global outcome many years after surgery even if not reflected on the EQ-5D and UPDRS II scores. In addition, there was also no significant worsening of the QOL and functional scores one year after surgery despite the progressive nature of the disease. This means that DBS still had a relative positive impact on QOL and functional outcomes one year after surgery in this real-life patient cohort though understandably less pronounced than what was seen in the more carefully selected cohorts in randomized trials. Although the CGIS was completed for most of the patients, the scoring was done retrospectively by our investigators exploiting data from patients' charts. The scoring system relied on documentation made by the first-hand clinicians, a method that has not been validated in other studies. The effect on caregiver burden was also not addressed. Finally, we did not correct for multiple comparisons due to the exploratory nature of the study and since we were looking at predetermined predictors prior to data collection [23]. Still, the relatively large number of comparisons in absence of such correction may have confounded the results to some degree; therefore the results of our study should be interpreted with caution in view of the limitations related to sample size and methodology. Overall, the majority of the significant predictors in our study conform to prior DBS literature which increases the confidence in those results. Our novel significant predictors like BMI will need validation in other cohorts.

In conclusion, our study suggests that certain disease characteristics may influence outcomes after DBS. While the majority of the patients in our cohort were globally rated as significantly improved on global scales, falls and balance dysfunction, absence of tremors, presence of dyskinesia, freezing of gait, and preoperative motor severity as represented by UPDRS III/MDS-UPDRS III were the most influential predictors of poorer outcome. In addition, some previously underrecognized patient characteristics may also influence DBS outcomes such as higher preoperative BMI and lower socioeconomic status. By confirming known DBS outcome predictors and identifying new factors, we hope to provide new insights into the process of patient selection and risk stratification prior to DBS. Further prospective studies utilizing higher number of patients and combining both objective and subjective outcome measures should be performed to confirm or refute the results of our study.

## Figures and Tables

**Table 1:** Beta estimates for health status measures collected at 2 follow-ups.

		6 months postop.			1 year postop.		
		*N*	Estimate (95% CI)	*P* value	*N*	Estimate (95% CI)	*P* value
UPDRS II	Age	39	−0.05 (−0.34, 0.23)	0.7058	32	0.03 (−0.3, 0.36)	0.8438
	Disease duration	38	−0.45 (−1.00, 0.11)	0.1127	31	0.21 (−0.56, 0.97)	0.5859
	BMI	37	0.49 (0.04, 0.94)	**0.0332**	32	0.68 (−0.002, 1.37)	0.0507
	Laterality (versus unilateral)						
	Staged bilateral	39	1.16 (−4.58, 6.89)	0.6849	32	0.53 (−6.75, 7.82)	0.8819
	Simultaneous bilateral	39	−3.68 (−12.69, 5.32)	0.4118	32	−5.46 (−14.34, 3.42)	0.2179
	Electrode passes (right)	29	2.09 (−0.41, 4.60)	0.0978	24	1.96 (−1.77, 5.69)	0.2876
	Electrode passes (left)	35	−1.21 (−4.29, 1.87)	0.4300	30	−2.18 (−6.14, 1.78)	0.2681
	Electrode passes (total)	36	0.31 (−1.15, 1.76)	0.6731	30	0.00 (−1.96, 1.97)	0.9963
	% equivalent levodopa dose	37	1.83 (−3.93, 7.58)	0.5233	30	0.89 (−6.15, 7.94)	0.7967
	On UPDRS III	39	0.09 (−0.21, 0.38)	0.5535	31	0.22 (−0.10, 0.54)	0.1709
	Tremor	39	−0.95 (−5.78, 3.88)	0.6909	32	1.35 (−4.41, 7.12)	0.6345
	Dyskinesia	39	−2.46 (−7.28, 2.36)	0.3072	32	−2.06 (−7.86, 3.74)	0.4736
	Freezing	39	2.32 (−2.97, 7.62)	0.3792	32	3.85 (−2.55, 10.26)	0.2284
	Falls/balance	39	6.48 (1.11, 11.84)	**0.0193**	32	6.45 (−0.38, 13.28)	0.0634
	Marital status	38	−1.86 (−7.35, 3.63)	0.4958	31	2.74 (−3.88, 9.36)	0.4041

EQ-5D index	Age	45	0.00 (−0.01, 0.00)	0.3403	36	0.00 (−0.01, 0.01)	0.9897
	Disease duration	43	0.01 (0.00, 0.02)	0.1810	35	−0.01 (−0.02, 0.00)	0.0696
	BMI	45	0.001 (−0.009, 0.011)	0.8450	36	−0.002 (−0.014, 0.011)	0.7983
	Laterality (versus unilateral)						
	Staged bilateral	44	0.08 (−0.02, 0.19)	0.1252	36	−0.01 (−0.14, 0.12)	0.9127
	Simultaneous bilateral	44	0.13 (−0.04, 0.29)	0.1351	36	0.003 (−0.15, 0.16)	0.9654
	Electrode passes (right)	33	−0.02 (−0.06, 0.02)	0.3494	28	−0.03 (−0.09, 0.04)	0.3837
	Electrode passes (left)	38	0.04 (−0.02, 0.11)	0.2006	32	0.07 (0.00, 0.14)	0.0508
	Electrode passes (total)	41	0.02 (−0.01, 0.05)	0.2458	34	0.01 (−0.03, 0.04)	0.6988
	Equivalent levodopa dose	42	−0.07 (−0.19, 0.04)	0.2000	34	0.06 (−0.06, 0.19)	0.3138
	On UPDRS III	44	−0.01 (−0.01, 0.00)	**0.0050**	35	0.00 (−0.01, 0.00)	0.6310
	Tremor	44	0.03 (−0.07, 0.13)	0.5516	36	0.07 (−0.03, 0.17)	0.1602
	Dyskinesia	44	−0.01 (−0.11, 0.09)	0.8448	36	0.02 (−0.09, 0.12)	0.7197
	Freezing	44	−0.03 (−0.13, 0.08)	0.5955	36	−0.09 (−0.19, 0.01)	0.0762
	Falls/balance	44	−0.06 (−0.17, 0.05)	0.269	36	−0.12 (−0.23, −0.02)	**0.0191**
	Marital status	44	−0.04 (−0.15, 0.08)	0.4992	35	−0.04 (−0.16, 0.07)	0.4668

**Table 2:** Patient Global Impression of Change Scale (PGIS) and Clinician's Global Impression of Change Scale (CGIS) results by various categorical predictors.

	% PGIS much improved or very much improved (proportion)	*P* value	% CGIS much improved or very much improved (proportion)	*P* value
Tremor	76.9% (20/26)	0.7566	84.0% (42/50)	**0.0075**
No tremor	82.1% (32/39)		60.0% (42/70)	

Dyskinesia	83.7% (36/43)	0.5167	64.2% (52/81)	**0.0380**
No dyskinesia	75.0% (18/24)		83.7% (36/43)	

Freezing	78.8% (26/33)	0.7591	61.9% (39/63)	**0.0315**
No freezing	82.4% (28/34)		80.3% (49/61)	

Falls/balance	75.0% (21/28)	0.3688	66.1% (41/62)	0.3218
No falls/balance	84.6% (33/39)		75.8% (47/62)	

Left unilateral	75.0% (6/8)	1.0000	63.6% (7/11)	1.0000
Right unilateral	77.8% (14/18)		67.6% (23/34)	

Unilateral	76.9% (20/26)	0.7906	66.7% (30/45)	0.7741
Two-stage bilateral	81.5% (22/27)		73.1% (38/52)	
Unstaged bilateral	85.7% (12/14)		73.1% (19/26)	

**Table 3:** Studies of functional, QOL, and global impression outcomes after DBS in PD.

Study	Functional, QOL, or global impression scale	Significant predictors	Number of patients	Time lapse since surgery
Welter et al., 2002	UPDRS II	(i) Age (ii) Disease duration (iii) UPDRS III (iv) Axial motor score (v) LED	41	6 months

Daniels et al., 2011	PD-Q39 SF-36	(i) Daily off time (+ve) (ii) Lower dyskinesia score (+ve) (iii) Improvement in UPDRS III (+ve) (iv) Improvement in psychiatric scales (+ve) (v) Reduction of dyskinesia (−ve)	61	6 months

Soulas et al., 2011	PD-Q39 SF-36	(i) Age (ii) Disease duration (iii) Depression (iv) Less use of social support coping	41	6 months and 12 months

Smeding et al., 2011	PDQL	(i) L-dopa response at baseline (+ve)	105	12 months

Maier et al., 2013	Subjective perceived outcome	(i) Depression (ii) Apathy	30	3 months

Floden et al., 2014	PD-Q39	(i) Depression (ii) Single-trial learning (episodic memory) (iii) Preoperative PD-Q39 score (iv) Bilateral surgery (+ve)	85	8 months (average)

Genc et al., 2016	MDS-UPDRS II EQ-5D CGIS	(i) Household median income	125 (43 for MDS-UPDRS II and EQ-5D)	6 months and 12 months

Maier et al., 2016	Subjective perceived outcome	(i) Apathy (ii) Axial motor score	28	12 months

Abboud et al.	MDS-UPDRS II EQ-5D PGIS CGIS	(i) Falls/balance dysfunction (ii) Dyskinesia (iii) Absence of tremors (iv) Freezing (v) UPDRS III (vi) Preoperative BMI	130 (45 FOR MDS-UPDRS II and EQ-5D)	6 months and 12 months

UPDRS II: Unified Parkinson's Disease Rating Scale, part 2: activities of daily living; LED: L-dopa equivalent dose; PD-Q39: Parkinson's Disease Questionnaire-39; SF-36: 36-Item Short Form Health Survey; PDQL: Parkinson's Disease Quality of Life Questionnaire; MDS-UPDRS II: Movement Disorders Society-Unified Parkinson's Disease Rating Scale, part 2: motor experience of daily living; EQ-5D: European Quality of Life 5-dimension Questionnaire; CGIS: Clinician's Global Impression of Change Scale; PGIS: Patient Global Impression of Change Scale.
